# Heterogeneity in pharmacological treatment and outcomes in Crohn’s disease patients in Catalonia: a population-based observational study

**DOI:** 10.1080/07853890.2022.2069851

**Published:** 2022-05-02

**Authors:** Eduard Brunet, Emili Vela, Luigi Melcarne, Laura-Patricia Llovet, Anna Puy, Montserrat Clèries, Caridad Pontes, Pilar García-Iglesias, Albert Villòria, Gilaad G. Kaplan, Xavier Calvet

**Affiliations:** a Servei Aparell Digestiu, Hospital Universitari Parc Taulí, Sabadell, Spain; b Departament de Medicina, Universitat Autònoma de Barcelona, Bellaterra, Spain; c CIBERehd, Instituto de Salud Carlos III, Madrid, Spain; d Unitat d’Informació i Coneixement. Servei Català de la Salut, Generalitat de Catalunya, Barcelona, Spain; e Digitalization for the Sustainability of the Healthcare System (DS3), Sistema de Salut de Catalunya, Barcelona, Spain; f Gerència del Medicament. Servei Català de la Salut, Barcelona, Spain; g Departament de Farmacologia, de Terapèutica i de Toxicologia. Universitat Autònoma de Barcelona, Bellaterra, Spain; h Departments of Medicine and Community Health Sciences, University of Calgary, Calgary, Alberta, Canada

**Keywords:** Epidemiology, inflammatory bowel diseases, Crohn’s disease, biologics, surgery

## Abstract

**Background:**

Heterogeneity in the treatment of a disease is a marker of suboptimal quality of care. The aim of this study is to evaluate the heterogeneity in the treatment used and the outcomes for Crohn’s disease (CD) in Catalonia.

**Methods:**

All patients with CD included in the Catalan Health Surveillance System (data on more than seven million individuals from 2011 to 2017) were identified. The different Catalonian health areas were grouped into 19 district groups (DG). Treatments used rates (systemic corticosteroids, non-biological and biological immunosuppressant) and outcomes rates (hospitalization and surgery) were calculated.

**Results:**

The use of systemic corticosteroids presented a decreasing trend over the study period, with an average rate of use in the different territories between 11% and 17%. The use of non-biological immunosuppressant treatment has remained stable, with an average rate of use ranging from 22% to 40% per year depending on the DG. The use of biological immunosuppressant treatment increased with an average rate of use in the different territories ranging from 10 to 23%.

Hospitalizations for any reason showed an increasing trend between 2011 and 2017 with an average rate of between 23% and 32% per year depending on the area. Hospitalizations for CD presented a decreasing trend, with an average rate of between 5% and 11% per year. Surgical treatment remained stable over time, rates per year were between 0.5% and 2%.

**Conclusion:**

A remarkable geographical heterogeneity in the use of different treatments and in outcomes of CD was observed between different geographical areas of Catalonia.

**KEY MESSAGE**
- There is a notable geographical heterogeneity in the administration of biological and immunosuppressive treatments to Crohn’s disease patients in Catalonia.
- There is also a geographical heterogeneity in their rates of hospitalization and surgical intervention.

## 1. Introduction

Inflammatory bowel disease (IBD), which includes ulcerative colitis (UC) and Crohn’s Disease (CD) [1], affects more than five million people worldwide [2]. The incidence and prevalence of CD are increasing progressively, especially in Western Europe and North America where prevalence is forecasted to approximate 1% of the population by 2030 [2–4].

First approved for CD in 1998, biological treatments have probably changed its natural history [5]. Their use has expanded quickly and multiple effective drugs are available today: anti-tumor necrosis factor (infliximab, adalimumab), anti-integrin (vedolizumab) and anti-interleukin 12/23 (ustekinumab) [6–10]. This variety of treatments may favour heterogeneity in CD management [11–14].

Many studies have shown a notable geographical heterogeneity in the management of IBD [15–17]. High variations have been shown in the use of anti-TNF drugs in IBD patients in the US [18]. In a prospective study in North America and Canada in children, Krishnakumar et al. observed that the proportion of children treated with thiopurines ranged from 6% to 89%, and reported that rates of children receiving corticosteroids ranged from 28.6% to 96.9% [19]. Recently, Zaltman et al. published a cross-sectional multicentre study observing also marked regional variations in IBD management in Brazil [20].

In CD, differences in management have been described not only between countries but also between hospitals [19,21–23]. They can be evaluated on the basis of the rates of patients receiving specific treatments. These differences may influence patients’ outcomes: for example, whether they undergo surgery, or whether they are hospitalized [24]. Regarding disease outcomes, epidemiological studies have also reported notable differences in surgery rates between European countries [25–27], which were also observed between Brazilian regions in a recent study [20].

As heterogeneity is a well-accepted marker of suboptimal care [28], detecting and analysing variability may help to improve care quality. The aim of this study was to evaluate the heterogeneity in the use of the most frequent medical drugs for CD (corticosteroids, non-biological immunosuppressive and biological treatments) and its relationship with CD outcomes in the health districts of Catalonia, using administrative data from the Catalan Health Surveillance System (CHSS).

## 2. Material and methods

### 2.1. Data source and study design

The regional health department of Catalonia, named CatSalut, provides universal healthcare coverage to all residents and collects detailed information on healthcare usage, including information from the minimum basic data set registered by healthcare units (e.g. hospitals, primary care centres, nursing facilities and mental health centres). CatSalut also collects information on drug prescription and billing for services (e.g. outpatient visits to specialists, emergency department visits, non-urgent medical transportation, outpatient rehabilitation and dialysis).

In 2011, the CHSS was created to integrate most of those activity registers, placing the patient (instead of the provider) in the centre of this information system, providing a more holistic and transversal view of health problems.

CHSS includes all the diagnoses reported by the different providers, regardless of whether they were recorded as the primary or secondary diagnosis. This information system collects all information from the entire public health system, including all hospital admissions and healthcare visits. Its automated data validation system checks the consistency of the data and identifies potential errors. Information from private health centres was not available [29].

All patients with CD included in the CHSS within the period 2011 to 2017 were identified, and classified according to the ICD-9-CM codes for CD. The ICD-9 codes used are shown in Supplementary Table 1.

**Figure 1.**
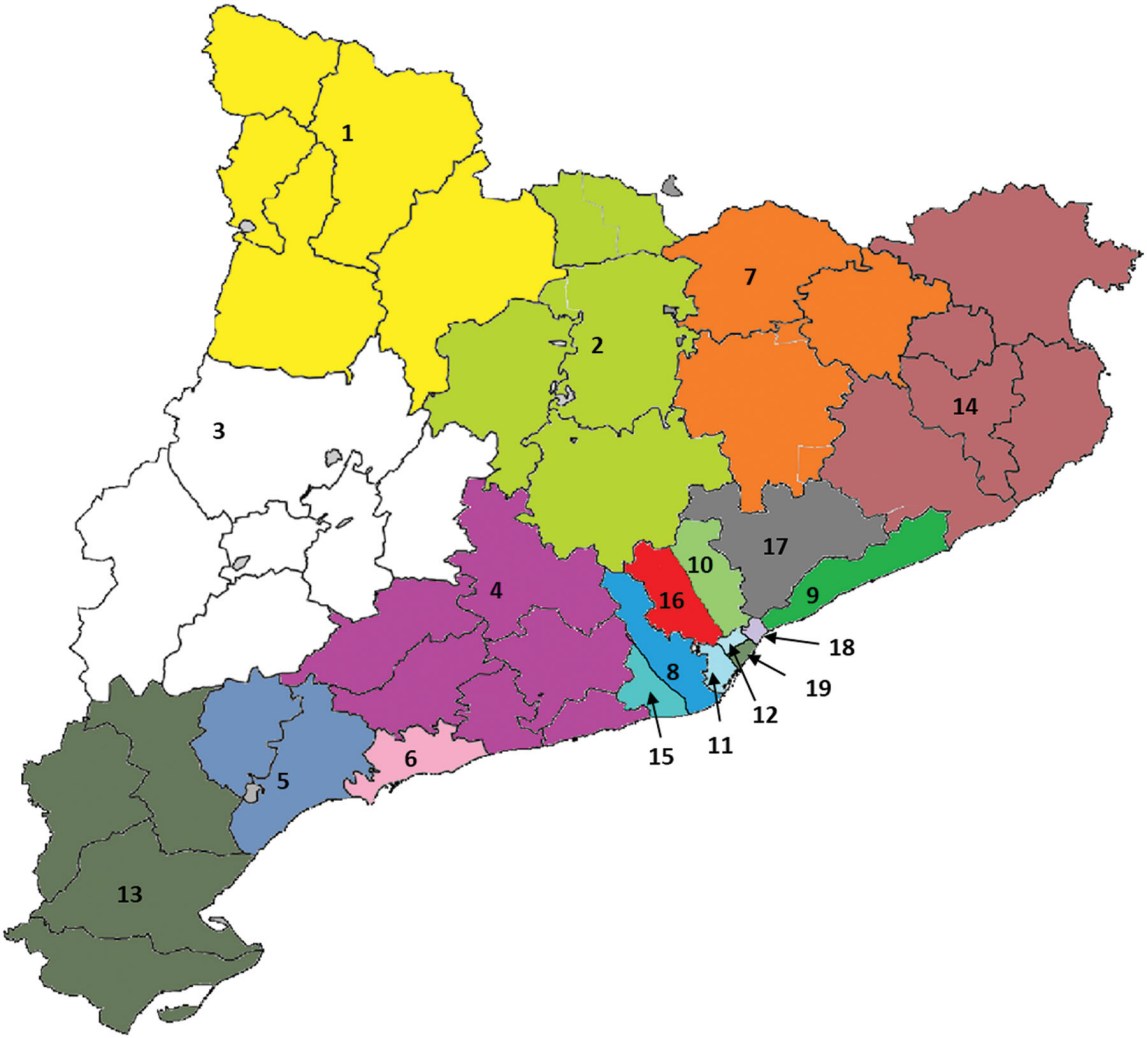
Geographical areas considered in the analysis.

For this study, the 43 health districts in Catalonia [30] were grouped into 19 Health Areas (HA) according to geographical proximity, setting (rural or urban) and reference hospital (Figure 1 and Supplementary Table 2). The assignment of patients to HAs was based on their home address and assignation to primary care. To allow comparisons, the HAs were designed so that each one would contain a similar number of individuals.

The exposure to different CD treatments was retrieved from the electronic prescription records for the same period. CD treatments included biological drugs (infliximab, adalimumab, golimumab – compassionate use for CD – vedolizumab and ustekinumab), non-biologic immunosuppressive drugs (azathioprine, 6-mercaptopurine and methotrexate) and corticosteroids. The list of active principles included in each pharmacological group is appended as Supplementary Table 3. Patients were considered to have been treated with a particular biological drug in a given year if they received at least one dose of the drug during this period. As a sensitivity analysis, we also measured exposure by dividing the total number of drug vials administered throughout the study period by the number of patient/years at risk.

Rates of hospitaliZation, CD-related hospitalization, surgeries and ostomies per 1000 patients/year were also obtained from the CHSS according to previously described criteria [31,32]. ICD-9-CM codes for the different surgical procedures are detailed in Supplementary Table 4.

### 2.2. Statistical methods

All data were calculated for each year, so as to estimate the evolution over the study period, and for the total study period so as to allow comparisons between HAs.

To calculate the rates of surgical procedures and drug exposure, first we calculated the time at risk for each patient. The time at risk began on 1 January 2011 (or at the date of diagnosis for incident patients) and ended on 31 December 2017 (or the patient’s death).

To estimate drug exposure, we used two different methods:

1. We divided the total number of patient/years with a given treatment during the study period by total patient/years at risk. Final rates were expressed as the number of patients treated with each drug for 100 patients/year.
2. As a sensitivity analysis, we also divided the total dispensations for each drug during the study period by the total number of patient/years at risk. Final rates were expressed as number of prescriptions of each drug for 100 patients/year.

To estimate the patient/year surgical rates, we divided the total number of ostomies and bowel resections during the study period by their time at risk. Final rates were expressed as ostomies or surgeries for 100 patients/year. To estimate the hospitaliZation rates, we divided the total number of hospitalizations during the study period by total time at risk. Final rates were expressed as hospitalizations for 100 patients/year.

Qualitative data are given as percentages and 95% confidence intervals and quantitative data as means ± S.D. Annual and geographical variation of treatments, hospitalizations and surgeries, were estimated with Poisson regression, adjusted by age, sex and socioeconomic status. The socioeconomic status was stratified into four categories of pharmaceutical co-payment: very low (recipient of social rescue aids), low (annual income < €18,000), moderate (annual income €18,000 to €100,000) and high (annual income > €100,000). Data on drug use and outcomes were displayed graphically as a colour gradient on a map of Catalonia. The study was performed and reported in accordance with the STROBE and RECORD Statement guidelines [33,34].

### 2.3. Ethical issues

The research used retrospective anonymized data from the CHSS. No personal data were used, and all the patient data were encrypted, so that no personal identification was retrievable or traceable to the original source from the working database. The study complied with the ethical guidelines of the Declaration of Helsinki. The study was reviewed and approved by the local ethics committee of the Hospital Universitari Parc Taulí in Sabadell (CEIC 2020/749, 24 November 2020). Given the retrospective nature of the study, the unavailability of personal data, and the impossibility of obtaining informed consent for the whole study population, the ethics committee waived the need for informed consent.

## 3. Results

**Figure 2.**
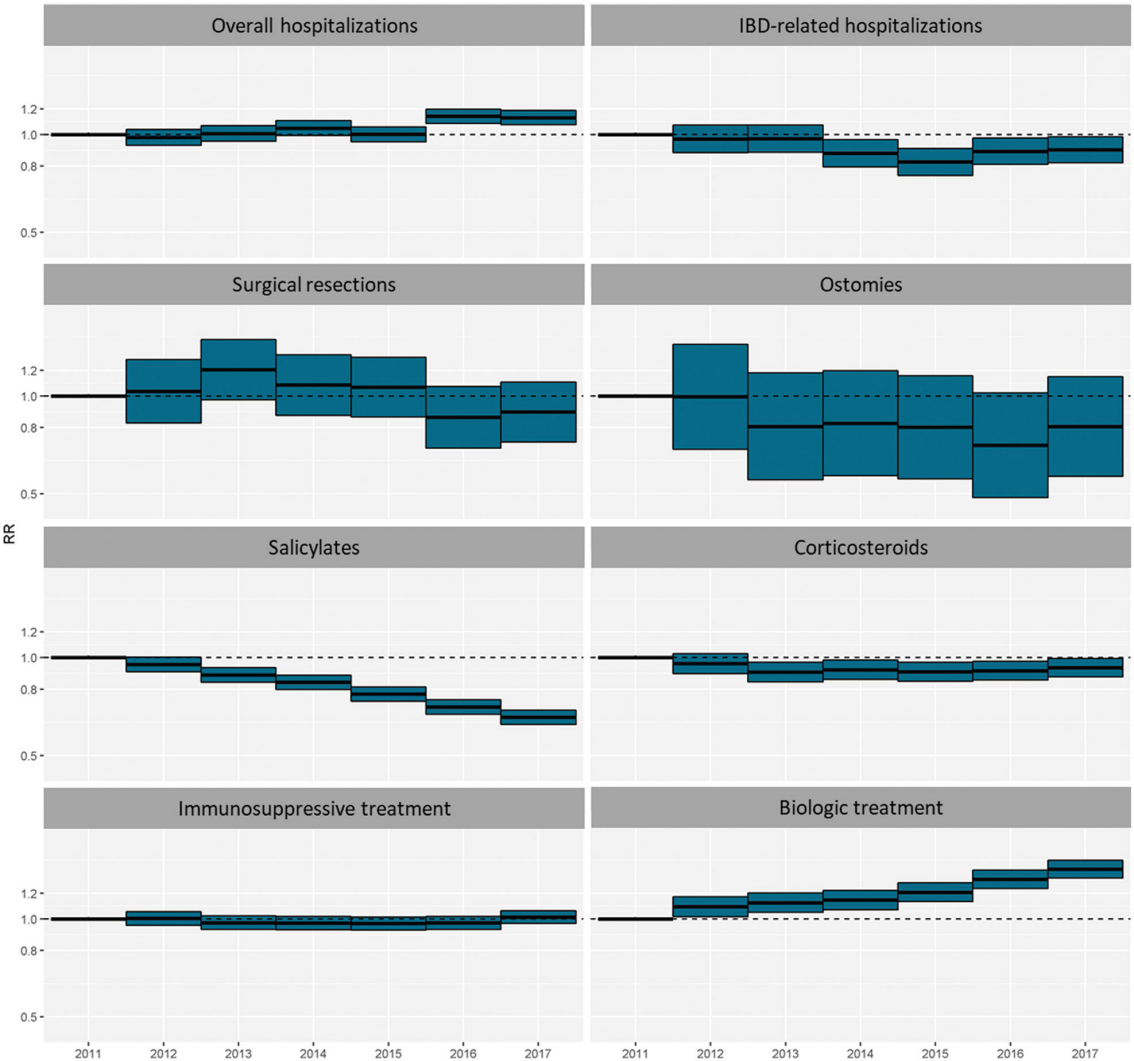
Relative risk of treatments and outcomes between 2011 and 2017.

As shown in our previous study [31,32], relative risk of overall hospitalization increased when compared with 2011, CD-related hospitalization rates slightly decreased (being statically significant in 2016 and 2017) and surgery and ostomies tend to decrease but not significantly. Regarding drugs, biological drugs increased (being statically significant over the period), immunosuppressant’s remained stable and salicylates and systemic corticosteroids significantly decreased (being statically significant between 2013 and 2017) (Figure 2, Supplementary Table 5).

### 3.1. Treatment heterogeneity

#### 3.1.1. Systemic corticosteroids

**Figure 3.**
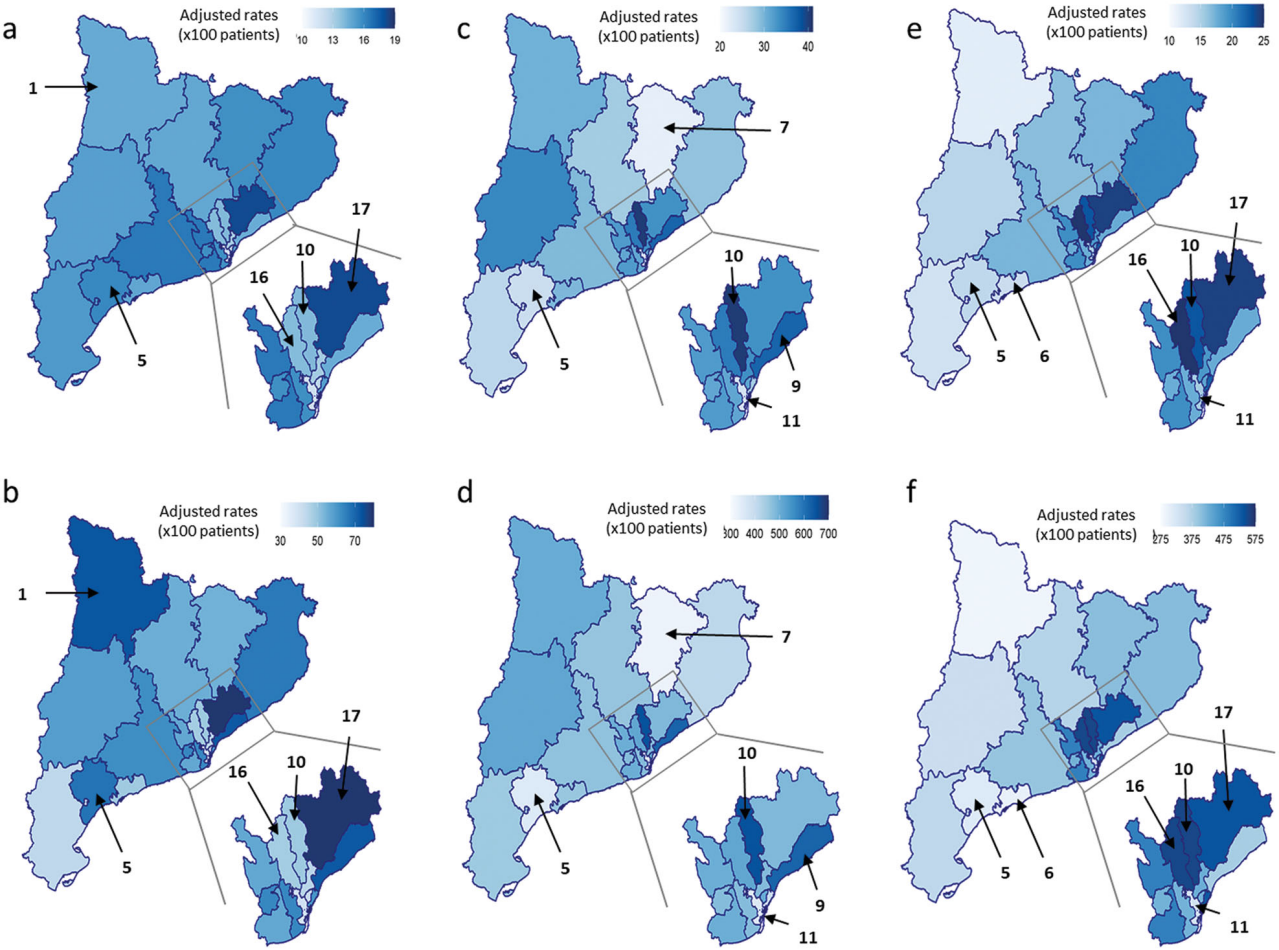
Variability in the use of medical treatments between HAs over the study period (2011–2017). (a) Mean annual rate of corticosteroid use per 100 patients. (b) Corticosteroid dispensation. Rate per 100 patients. (c) Mean annual rate of immunosuppressive treatment per 100 patients. (d) Immunosuppressive treatment dispensation. Rate per 100 patients. (e) Mean annual rate of biological treatment per 100 patients. (f) Biological treatment dispensation. Rate per 100 patients.

The use of systemic corticosteroids tended to fall over the study period, with a mean annual rate of use per 100 patients in the different HAs ranging from 11% to 17% (Figure 3(a)). The prescription rate remained fairly unchanging for each HA over time (Supplementary Table 6 and Supplementary Figure 1).

**Figure 4.**
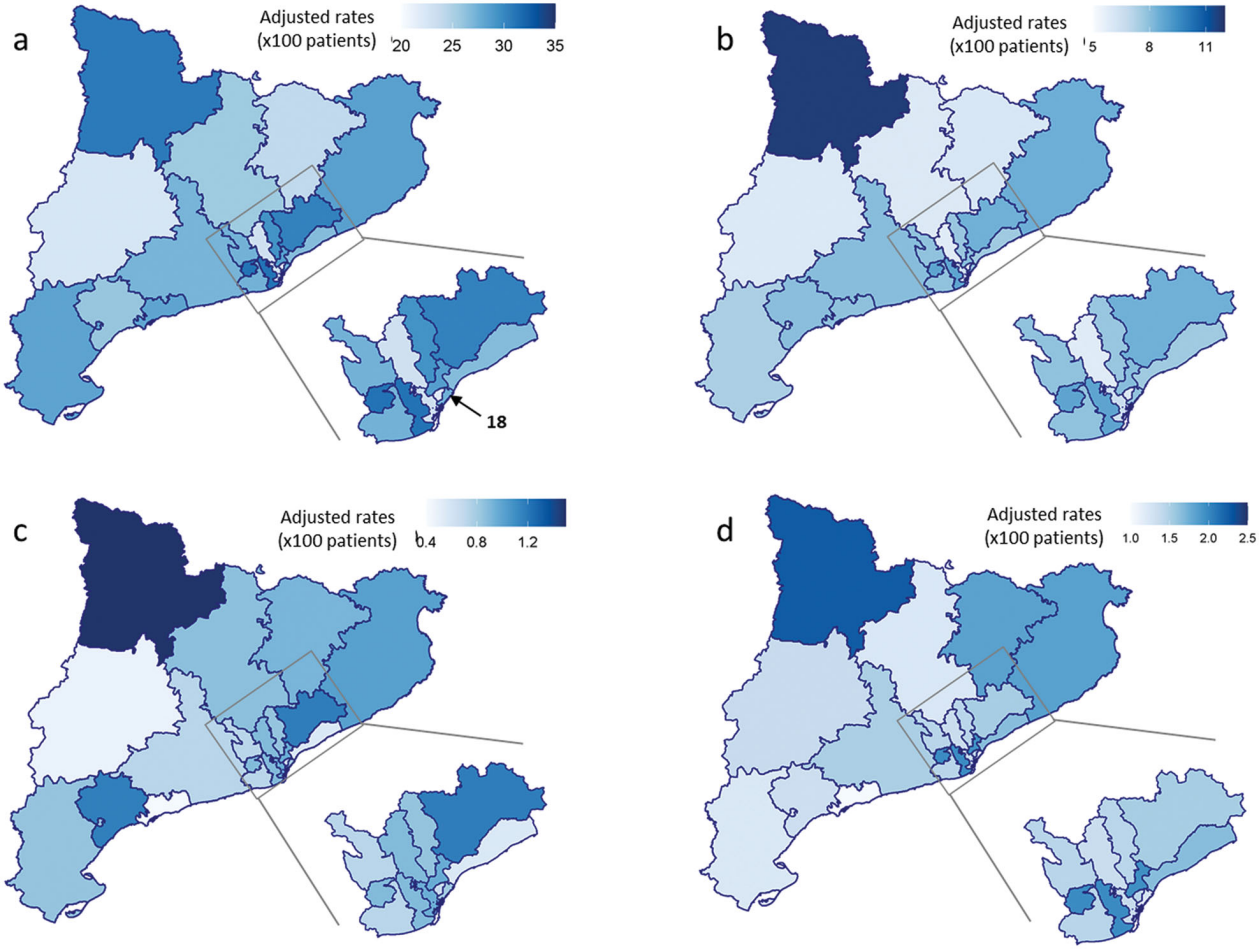
Variability in the outcomes between over the study period (2011–2017). **(a)** Mean annual rate of overall hospitalization per 100 patients. (b) Mean annual rate of IBD-related hospitalization per 100 patients. (c) Mean annual rate of ostomies per 100 patients. (d) Mean annual rate of surgical resections per 100 patients.

Sensitivity analysis showed big differences between the rate of patients treated with corticosteroids each year and the total dispensations doses for 100 patients (Figure 3(a,b) and Supplementary Table 7). Thus, some areas had treated a small amount of patients with a low number of total doses (HA 10 presented an average rate of use of 13.1% and HA 16 of 11.9%), thus suggesting overall reduction in the use of steroids. By contrast, other areas showed a medium-range number of patients treated but with a large number of prescriptions (HA 1, with a 15.9% of use). The fact that many prescriptions were used in a relatively small number of patients reflects an extensive use of steroids, strongly suggesting that many patients had received steroids in long courses or even as maintenance treatment. Finally, one specific HA (HA 17 with a 16.3% of use) presented a marked increase in both the number of patients treated and the number of prescriptions, thus indicating a generalized overuse (Figure 3(a,b)). In most areas, high steroid use was associated with a trend towards poorer outcomes (Figure 4) and a fall in the use of immunosuppressive and biological treatments (Figure 3c–f).

#### 3.1.2. Non-biological immunosuppressive treatment

The use of non-biological immunosuppressive treatment remained steady between 2011 and 2017, with a mean rate ranging from 22% to 40% per year depending on the HA (Figure 3(c, d)). Some HA’s presented a significant and elevated use of this treatment (HA’s 9 and 10 with a 36.1% and 39.3% of use respectively), whereas others had significantly lower rates of use compared with the whole population (HA’s 5, 7 and 11 with a 23.3%, 21.3% and 21.4% of use respectively). Detailed data for HA and year are shown in Supplementary Table 8 and Supplementary Figure 1.

As a chronic treatment, the sensitivity analysis did not show marked differences (Supplementary Table 9).

#### 3.1.3. Biological treatment

The use of biological treatment tended to increase over the study period. The mean rate of use in the HAs ranged from 10% to 23% (Figure 3(e,f)). Some HA’s presented a significant and sustained increase in the use of this treatment (HA’s 10, 16 and 17 with a 21.5%, 20.5% and 22.1% of use respectively), but others HA’s presented a significant and sustained reduction (HA’s 5, 6 and 11 with a 12.2%, 11.9% and 12.2% of use respectively). Detailed data for HA and year are shown in Supplementary Table 10 and Supplementary Figure 1.

As a chronic treatment, the sensitivity analysis did not show marked differences (Supplementary Table 11).

### 3.2. Hospitalization rates

The mean total hospitalization rate rose from 23% in 2011 to 28% in 2017 (Figure 3). The mean rate ranged from 22% to 32%, depending on the HA: the lowest rate of total hospitalization was found in HA 18. In contrast, CD-related hospitalizations remained constant over time, ranging between 8% in 2011 and 7% in 2017 (Figure 4(a,b)). The rate of CD-related hospitalization ranged from 5% to 11% between the different HA’s, but no sustained trends were observed. Detailed data for HA and year are shown in Supplementary Tables 12 and 13 and Supplementary Figure 2.

### 3.3. Surgery rates

Surgical treatment (both resections and ostomies) remained unchanging over time. Rates per year were low, between 0.5% and 2% (Figure 4(c,d)). There were no significant differences between HAs. Detailed data for HA and year are shown in Supplementary Tables 14 and 15 and Supplementary Figure 3

### 3.4. Correlations between treatment use and outcomes

A significant positive correlation was observed between both total and CD-related hospitalizations and corticosteroid use (0.53; *p =* 0.01 and 0.45 *p* = 0.05, respectively). The correlation was non-significant for non-biological immunosuppressive treatments (0.03; *p =* 0.88 and 0.16; *p =* 0.49 respectively) and for the use of biological treatment (−0.19; *p =* 0.43 and −0.16; *p =* 0.51 respectively).

The correlation between the use of corticosteroids and the two surgical procedures, ostomies and resections, had a positive estimate that did not reach signification (0.33; *p =* 0.16 and 0.25; *p =* 0.28 respectively). Biological agents showed a non-significant negative correlation estimate with ostomies and resections (−0.18; *p =* 0.45 and −0.12; *p =* 0.62 respectively), as did the use of non-biological immunosuppressive drugs with ostomies (−0.22; *p* = 0.35). (Detailed data on correlations are shown in Supplementary Table 16 and Supplementary Figure 4).

## 4. Discussion

Data on the prevalence, incidence and mortality on CD and demographical variables have been described in detail in previous articles [31,32].

This study shows that despite there is heterogeneity in the pharmacological treatment and the outcomes of CD patients in Catalonia (the differences may be as high as 200% in the use of biological drugs or in the rates of surgery), these differences, are not as high as those reported in other settings. For example, the differences in the use of biological drugs in the US were found to range from less than 5% to more than 40% depending on postal code [35]. The differences may depend on the availability of specialized care or IBD units. Thus, in the US, Ananthakrishnan et al. observed much lower differences in the use of biologicals when comparing seven high volume centres [36]. The variability, however, seemed to persist, even between very specialized centres. This and other studies [16,17,20] suggest that there is a significant variation in the management of CD disease even between experts.

Rates of variability may depend on the equilibrium between factors that favour the standardization of management and those that may induce heterogeneity. In our opinion, there were two factors that reduce the variability to the moderate (though significant) rates we observed. First of all, the presence of a widespread and relatively homogeneous public healthcare structure may help to reduce heterogeneity. Thus, our study found similar rates of heterogeneity to those observed in other countries such as Korea and Canada. In the three main cities in Korea (Seoul, Deagu and Busan), Han et al. found rates of biological treatment use of 20.7%, 22.9% and 14.6% respectively [37]. Second, GETECCU, the Spanish Crohn’s disease and ulcerating colitis working group has been very active in training IBD specialists [38] in developing standards of care [39], and in accrediting IBD units according to these standards [40]. Although these homogeneizing influences have probably reduced variability, geographical differences remain an issue. In a Canadian population study, Kuenzig et al. observed that patients from rural zones were less likely to be treated by a specialist gastroenterologist and presented a higher rate of comorbidities and infectious diseases than patients from urban areas [41]. Even in a smaller territory like Catalonia, where distances to acute hospital care is in average less than 20 Km [42], the access to IBD specialists and IBD units differs in rural and urban areas. Indeed, our study described non-significant lower rates of use of biological drugs and higher ostomy and surgery rates in patients living in rural areas located far from a major hospital.

Most correlations between treatment and outcomes in our study are non-significant. This finding may have many different (and possibly complementary) explanations. First, as the variability in medical treatment is moderate, using treatments between the observed ranges may have had a limited effect on outcomes. Second, other factors such as the availability of surgery, patients’ or medical team’s preferences or other structural factors (for example, access to a day care unit for early treatment of flares) may also play a role in deciding the choice of hospitalization or surgery, which may be even more important than pharmacological treatment. The only significant correlation observed was between corticosteroid use and both total and CD-related hospitalizations. There is a global trend to decrease the use of corticoids that have been progressively substituted by safest therapies [32]. The current study, however, shows that there is still a high rate of steroid overuse in some areas. The increased rate of hospitalization associated to steroids may have different possible explanations: Steroids may be related, per se, to a poorer control of the disease or more adverse events leading to hospitalization or, alternatively, a high rate of corticosteroid use may be a marker for a more general situation of suboptimal care – for example, unavailability of timely on demand attention during flares – leading to an increase hospitalization rates.

This study has certain limitations. First, the CHSS database includes only patients who use the public health system. This means that, conceivably, some patients with mild disease might not have been recorded. However, the public system is used by over 80% of the population of Catalonia; the rate of use is, therefore, much higher, since the 20% of non-users also includes healthy individuals who also have access to the system in case of need. Furthermore, as CD is a chronic disease and pharmacological (and especially biological) treatments are relatively expensive, most patients request financial support from the public system. In very expensive treatments such as biologicals, public prescription approaches 100%. It is also important to consider that the tool to collect data on biological treatments by the CHSS database was not mandatory for invoicing until 2014. Until then, some biological treatments were invoiced as inhospital medications in certain sites. Thus, heterogeneity in biological treatments used before 2014 may be partly influenced by different speed in recording data amongst sites that were late uptakers of the voluntary registry. This fact, however, has probably no effect in the results. Although the use of biological treatments increased steadily between 2011 and 2017, the increase was greater between 2015 and 2017 when recording data on biological treatments were mandatory. A final limitation is that it is also known that patients with aggressive CD (i.e. penetrating or structuring patterns) need more aggressive treatment and have higher rates of biological treatments, hospitalization and surgery. Unfortunately, information on the baseline severity of the disease was not available for analysis. In addition, perianal-CD and UC was not included in the analysis due to their potential higher rate of misclassification using ICD-9-CM codes.

A plausible explanation of the lack of huge variations across the territory is that the organization of care allows derivation of cases, so that escalation of severe cases for care to tertiary sites with wider experience in both surgery and advanced medical care is foreseen and implemented. In Catalonia 98% of the population has a distance lower than 20 km to the closest acute care hospital, and roughly half of the population lives in the metropolitan area, where six high technology public hospitals are placed [43]. The organization of healthcare ensures that access to highly specialized care is granted to patients regardless of where they live in the territory.

The data provided by this study and by previous research may offer an example of a rather successful care of this disease. However, some measures may help to further improve treatment. For example, non-specialist physicians in rural areas would benefit from having access to IBD specialists by teleconference or e-mail. The results of the study may also increase the pressure to develop value-based medicine tools as, for instance, Care Improvement communities, which have shown their ability to quickly improve patients’ outcomes. For example, within a year of launch of the paediatric IBD ImproveCareNow project [44], the rate of children in remission increased from 77% to 83% [45]. Future studies should address the effect of heterogeneity in patient outcomes. It is also necessary to initiate measures like local guidelines, surveys, courses or conferences to unify the management of CD in our territory.

In conclusion, in this register-based, population-wide study, we observed a remarkable geographical heterogeneity in the use of different treatments and in the rate of hospitalizations and surgeries for CD between different geographical areas of Catalonia. Reducing this variability represents a clinical and organizational challenge, but is likely to increase equity and quality of care. In the same sense, it is essential to improve clinical records and standardize results measurement in order to carry out good-quality research.

## Data Availability

Data cannot be shared for ethical/privacy reasons. The data underlying this article cannot be shared publicly due to is individual date from a population registry from Catalan Health Surveillance System and only can be accessed from Catsalut. The data will be shared on reasonable request to the corresponding author.
